# Factors determining male introduction success and long-term stability in captive rhesus macaques

**DOI:** 10.1371/journal.pone.0219972

**Published:** 2019-07-17

**Authors:** Astrid Rox, André H. van Vliet, Elisabeth H. M. Sterck, Jan A. M. Langermans, Annet L. Louwerse

**Affiliations:** 1 Animal Science Department, Biomedical Primate Research Centre, Rijswijk, The Netherlands; 2 Animal Ecology, Department of Biology, Faculty of Science, Utrecht University, Utrecht, The Netherlands; 3 Department of Animals in Science and Society, Faculty of Veterinary Medicine, Utrecht University, Utrecht, The Netherlands; Institute of Animal Science, CZECH REPUBLIC

## Abstract

The entrance of new males into non-human primate groups bears high social risk, yet migration is necessary to prevent inbreeding. Males are not always accepted in their new group. In the wild, males may increase the likelihood of successful group entry by choosing a new group based on their own and the group’s characteristics. Understanding whether these characteristics also determine a male’s ability to enter captive groups is crucial to improve introduction management. This study aims to identify which factors determine male introduction success (i.e. male stays in the group for at least 4 weeks) and long-term stability (i.e. the male does not cause considerable behavioural problems after success) after male introductions in captive groups of rhesus macaques (*Macaca mulatta*), creating one-male groups. We studied 64 male introductions at the breeding colony of the Biomedical Primate Research Centre in Rijswijk, The Netherlands. 49 (77%) introductions were successful, with the male obtaining a long-term stable social position in the group in 38 (59%) introductions. Introductions of males that reached at least prime age, into groups with more adult females, but without pregnant females were most successful. Moreover, long-term stability was highest when males were heavier, were at least 3.5 years old when they were first removed from their natal group, and groups had few matrilines and no pregnant females were present. Males should be introduced at the time they would naturally immigrate, when they are strongest. Moreover, groups should consist of few large matrilines, as observed in the wild, with philoatric females and males that are removed at natural age. Our study highlights the importance of composing naturalistic groups and mimicking natural migration patterns to maintain long-term stable breeding groups in captivity.

## Introduction

Primates are highly social animals with the behavioural need to display a wide variety of complex social behaviours. The welfare of captive primates can be enhanced through naturalistic group housing, which will enable them to display natural social behaviour [1,2]. Naturalistic group housing is common in zoos, while primates in research facilities are generally housed in unnatural groups. Especially pair-housing is common in research facilities. It is therefore not surprising that much research has been done on the management of pair-housed individuals. It is known which animals should be introduced to each other during pair-formation, and which introduction technique is best [3–8]. However, introducing animals into larger and more naturalistic groups is considered more difficult and more risky than pair-formation, as primate groups have complex social dynamics. Yet, there is very limited knowledge on the management of naturalistic primate groups, while introducing new males is necessary to prevent inbreeding. The Biomedical Primate Research Centre (BPRC) in Rijswijk, the Netherlands is a research facility that houses macaques (*Macaca spp*.) in naturalistic one-male groups. Their primates live in groups wherein natural group composition and migration patterns are mimicked to optimize primate welfare.

Wild macaques live in multi-male multi-female groups with male biased migration to prevent inbreeding [9,10]. Groups generally consist of a few matrilines with multiple adult females, their non-adult offspring and several adult males. Females are phylopatric and remain in their natal group during their lives [2,11,12]. Males migrate from their natal group when reaching sexual maturity, and may change groups several times in their lives [2,10,13–16]. The arrival of a new male into an existing group leads to extensive male-male competition, commonly resulting in severe injury or sometimes even death [2,14–16], or unsuccessful immigration [13,17]. This male-male competition is often prevented in naturalistic captive groups through the formation of single-male multi-female (i.e. harem) groups. The resident breeding male is generally removed from these groups before a new male is introduced. Still, similar to male immigration in the wild, male introductions into captive multi-female groups are associated with high aggression levels and stress [18–20]. Moreover, males may fail to enter a group, leading to unsuccessful introductions ([20], records BPRC). Furthermore, even after successful group entry a wild macaque male may not be able to obtain a long-term stable position in his new group [13,21]. Similarly, successful introductions may lead to socially instable groups in captivity [4,18,20] where some individuals need to be separated. Thus, not only a male’s ability to enter a group initially is crucial for introduction management, also a male’s ability to obtain a stable social position in the group is highly important. Composing long-term stable groups should be a main goal of introductions, as animals in socially instable situations often suffer from stress and injuries through increased aggression levels [22–25]. Moreover, the frequency of risky introductions can be reduced when males maintain their position in a group for a longer period of time. In the wild, migrating males may increase the likelihood of successful group entry and obtaining a stable social position by timing their migration with the least female resistance, waiting for optimal body condition [10], and by preferably entering groups with the least resistance to new males. Applying knowledge obtained from the wild may increase introduction success and long-term stability.

First, the timing of male group entry may play a role. Male migration in seasonal breeding species (e.g. wild rhesus macaques, *Macaca mulatta*) is concentrated right before or early in the breeding season [26–33], as females may be more receptive to new males during this period. Captive male rhesus macaque introductions are indeed more successful during the breeding season, compared to outside the breeding season [20]. This implies that timing male introductions with the time frame of natural immigration may increase the chances of male group entry in captivity.

Second, male characteristics may affect a male’s ability to successfully enter a group. Single-male introductions into multi-female groups mimic the so-called bluff strategy observed in the wild [13,14,30,31,34,35]. Bluff immigrants attempt to obtain the alpha position directly after entering a new group. Sub-adult, juvenile, and old males are less successful as bluff immigrants than full-grown prime adult males [26,31,35,36]. In addition, males may not enter a new group before they reach high body mass. These heavier males are more successful in taking over a new group [10,37]. Taken together, this suggests that males at their prime age and males with higher body mass may be more successful during introductions than younger, older, or lighter males. However, not only a male’s strength may determine his ability to enter a group and maintain a social position, they will also need social skills. Males may gain social skills in their natal group. On average, male macaques leave their natal group at the age of almost 4, with variation from 3 to 8 years [26,27,32] and 90% of the males have left their natal group at an age of 6 years [27]. Removing animals too early from their social group in captivity may lead to inadequate social behaviour [38–40], thereby negatively affecting a male’s ability to settle in a new group. Additionally, males may gain social experience in non-natal breeding groups, since they may change groups several times in their lives [21,41,42]. Males may use their social experience gained in a previous group to increase chances of successful group entry during their second immigration. Therefore, captive males with experience as breeding male may be more successful and more long-term stable during introductions.

Third, the composition of the group may affect a male’s ability to enter a new group. Males likely enter groups with a composition that increases the chances of successful entry. Males may prefer new groups with a large number of females, as sexual attraction to females is the likely reason for males to migrate [16,29]. Yet female resistance may be larger in groups with more females, as female coalitions against new males may be larger [17,18,43]. Possibly, the males balance their preference for more females with the additional costs of increased female resistance. In line with this balance, the number of females does often not affect a male’s group choice [13,26,27,29,34,44]. In addition, females often form coalitions with their family members [45], and the presence of family is important to maintain group stability [11,46,47]. Groups with a large number of families (i.e. matrilines) may be more unstable, which is increased even further through introductions [11,46,48]. This indicates that introductions into groups with few females and few matrilines may be more successful. Female reproductive state may affect their attitude towards new males, and their tendency to join coalitions. Lactating and pregnant females can be at risk of infanticide when a new male enters their group [49–51], and may show more resistance to a new male [52,53]. Moreover, the presence of sub-adult males in a group may affect male group entry. Male long-tailed macaques (*Macaca fascicularis*) avoid immigration into groups with many sub-adult males [21], while immigrant crested macaques (*M*. *nigra*) often enter groups with more males [34]. In Japanese macaques (*M*. *fuscata*), young males preferred to enter groups with many other males, while older males preferred groups with few adult males [35]. Overall, the number of females and matrilines in a group, female reproductive state, and the presence of sub-adult males may affect a male’s group choice in the wild. Determining whether or how these factors also affect male introduction success and long-term stability is crucial to improve captive introduction management.

This study aims to identify the factors affecting male introduction success and long-term stability in captive rhesus macaques. We studied the effect of introduction timing, male characteristics and group characteristics during single-male introductions (i.e creating one-male groups) in the rhesus macaque breeding colony of the Biomedical Primate Research Centre in Rijswijk, the Netherlands. Firstly, we expect introductions right before or early in the breeding season to be more successful than introductions later in or outside the breeding season. Secondly, we expect prime aged males, males with higher body mass, more experienced males, and males who left their natal group at a natural age to be more successful during introductions. Lastly, introductions into groups with fewer females, fewer matrilines, without sub-adult males, without pregnant females, and with fewer lactating females may be more successful. We expect similar patterns for long-term stability.

## Methods

### Subjects

Data were collected on 64 male rhesus macaque introductions into existing breeding groups at the Biomedical Primate Research Centre (BPRC) in Rijswijk, The Netherlands. We defined existing breeding groups as groups with multiple adult females (age ≥3) and their non-adult offspring, who had already lived together with a breeding male prior to the studied introduction. Natural migration patterns are mimicked in these groups, as females remain in their natal group during their lives and males are generally removed when reaching sexual maturity. These removed natal males are housed in small (i.e. 2–8 individuals) all-male groups for several years, before they may be introduced as a breeding male. This form of naturalistic group-housing increases animal welfare and provides better animals for biomedical research, as it increases repeatability and reproducibility of the results [54], and thereby contributes to refinement and reduction.

The studied groups ranged in size from 4 to 32 animals (average: 15.8), with 2 to 16 adult females (average: 7.4) divided over 1 to 7 different matrilines (average: 3.0) in each group. 30% of the females (range: 0–80% per group) were lactating during the introductions (i.e. they had an infant aged <1 year at the start of the introduction). Pregnant females were present in 19 groups, varying from 1 to 6 pregnant females (average: 2.1) per group. Females were considered pregnant if they gave birth to an infant fathered by a male (i.e. previous breeding male or natal male) that was removed from the group before the introduction started. When a natal male that remained in the group during the introduction fathered a child, females were considered pregnant if they gave birth less than 4.5 months after the introduction started. Paternity was determined based on genetics [55]. These natal males (age ≥3) were present in 20 of the groups; with on average 1.75 (range: 1–4) natal males in these groups.

During the 64 introductions, 49 different males were introduced; 34 males were studied once, while 15 males were studied during introductions into two different groups. As only few males were used multiple times, male identity could not be included as a factor in our analysis. However, 3 out of 4 studied male characteristics change over time. Therefore, we considered multiple introductions of the same male as independent data points. The same is true for the groups the males were introduced to. Generally, introductions were separated in time by several years. Group composition, and thereby dynamics, changes over time. Individuals may be born into the group, may die or mature, or can be removed. Sometimes, groups are even split or female rank reversals take place. Groups sampled at different time points are therefore not comparable. Only once, a group experienced a male introduction twice in one year. However, several adult females causing trouble when introducing new males were removed from the group between the two introductions, changing the group composition and dynamics.

Experienced males were introduced in 42% (N = 27) of the cases. Males were considered experienced if they spent time as a breeding male in a social group prior to the studied introduction. The males’ ages varied from 4.25 to 22.16 years (average: 9.63), while the ages at which they were removed from their natal group varied from 0.38 to 9.17 years (average: 3.89). All animals born at the BPRC since 2001 are group-reared and left their natal group when they were at least 2.5 years old. All males had the opportunity to mature after being removed from their natal group. No males were directly transferred from their natal group to a new breeding group. All males were unfamiliar and unrelated to the groups they were introduced into.

The groups were housed in spacious inside and outside enclosures of approximately 3m high and measuring approximately 280m^2^ in total. The enclosures contained multiple environmental enrichment items, such as climbing structures, fires hoses, tires, and a swimming pool [56]. The enclosures could be separated into different compartments. During introductions, the group’s access to some of these compartments could be restricted. For a detailed description of the housing conditions during the introductions see Rox et al., 2018. The animals always had plenty of opportunity to avoid each other and they could make use of visual barriers.

### Introductory procedure

The studied introductions were management procedures carried out by experienced animal caretakers, between 2003 and 2016. 25 of the introductions started right before or early in the rhesus macaque breeding season (i.e. between September and December), while 39 introductions were started at the end of or outside the breeding season. The rhesus macaque breeding season at the BPRC lasts from October until March. All introductions followed the BPRC introduction guidelines, wherein males are familiarized with and introduced to the group stepwise. The experienced animal caretakers closely monitored the introductions. During the first steps, a male only spent time with the group under supervision. When the introductions progressed, the animal caretakers’ supervision gradually decreased up to the point no supervision was present. The animal caretaker decided whether supervision was needed and when the introduction could progress, based on personal knowledge and experience. The risk of severe aggression and the interest between the group and the new male were estimated.

Prior to an introduction, the previous breeding male was removed from the group, often together with all three year and older natal males. Generally, this took place approximately one year before the introduction. How long a group spent without a male depended on the presence of infants, the number of animals born in the breeding colony, and the genetic representation of the group in the breeding colony. Then, a familiarization phase started several weeks before an introduction. First, the male was moved into an inside compartment that separated him from the group through a concrete wall. This situation allowed auditory, olfactory, and minimal visual contact between the group and the new male. Next, the male was provided with full-visual, and limited physical contact with the group through wire mesh. This could last from a few hours up to a week. The next step depended on the behaviour of the animals. If aggression levels remained high for a longer period of time, the animal caretaker chose to familiarize a single female or a small group of females with the male. The male and the female(s) would spend a few hours up to two days in the same compartment of the enclose. Then, either a new (group of) female(s) was familiarized with the male, or the physical introduction started. If female aggression levels through the fence diminished quickly and the group was interested in the new male, the physical introduction was started immediately. During two of the introductions in our sample, aggression remained high during the familiarization period. These introductions were stopped and were classified as unsuccessful.

The first step of the physical introduction was introducing the male to the entire group in the outside enclosure. Generally, the male spent 1 to 1.5 hours with the females on the first day. This time was gradually increased as the introduction progressed. Next, additional access to the inside enclosure was provided when the male spent time with the group. Eventually, the male spent approximately 7 hours per day with the females. When the group appeared stable, the male remained in the group full-time. This occurred on average after 44 days (median: 12 days). However, the duration of the introductions varied from 1 to 357 days, with only six introductions exceeding a 100-day period. The exact timing could differ between the introductions, based on the animals’ behaviour. Some aggression during introductions is normal, and aggression levels are often elevated at the start of male introductions [18]. In general, conflicts between animals are brief, and are necessary to confirm their position towards unfamiliar individuals. The animal caretakers only interfered in aggression and stopped the introduction if severe aggression remained between the group and the new male, or the male or females were (at risk of being) severely injured during the introduction. Aggression could generally be stopped from outside the enclosure by either producing loud noise, opening and closing hatches in the enclosure, or pretending to open the door to the enclosure to enter. Then, males could be removed from the group, by moving them into a compartment wherein a concrete wall separates them from the group, Often, the males are happy to move away from the group after interfered conflicts. Therefore, males generally move to the separated compartment voluntarily. In two cases the introduction appeared to progress well for a few weeks, but the male was found dead due to female inflicted injuries.

### Data collection

Data were collected from the digital database of the BPRC. This database contains the main characteristics of each animal at the BPRC (e.g. gender, date of birth, etc.), and includes reports on management procedures (e.g. moving to new enclosure), childbirths, and genetic information (e.g. relatedness to other individuals). Moreover, the database contains information on all introductions, in the form of the summarized administration of the animal caretakers. The start date of each of the different familiarization and introduction steps, the end date of the introductions, and important behavioural events (e.g. female interest and severe aggression) were noted. We retrieved this information on all male introductions and determined the composition of the group a male was introduced to. We selected all male introductions into existing breeding groups at the current housing facilities of the BPRC breeding colony for our analysis. The raw data is available in S1 Appendix.

### Ethics statement

The introductions concerned management procedures necessary to prevent inbreeding at the BPRC breeding colony and were not experimentally induced. This study is a retrospective analysis of these introductions and only used information from introduction reports. The introductions adhered to all institutional, national and European animal welfare standards. No invasive research or experimental procedures requiring ethics approval according to the European Directive 2010/63 and the Dutch law were performed. Therefore, no approval by the BPRC animal ethics committee was required. Nevertheless, the management procedures were communicated with and approved by the institute’s Animal Welfare Body (IvD BPRC). Introductions were performed under supervision of an experienced ethologist and all animals were under close observation by one of the veterinarians of the BPRC.

### Measures

All measurements were calculated based on the start date of an introduction, defined as the first day a male was physically introduced into the entire group. The majority of variables included in our analysis were transformed into categorical variables, because either the expectations were non-linear or the variables did not meet the assumptions for our statistical models (see below: *Statistics*). Whether the introductions took place right before or early in the breeding season or not (early *versus* late), and whether the introduced males were experienced (experienced *versus* inexperienced) were already categorical factors. Male age at the start of the introduction was divided into three categories. Males between the age of 7 and 12 years were classified as *prime* aged males, based on data from free-ranging rhesus macaques [57] and the notion that animals in captivity generally mature faster than in the wild [58]. Males before their prime age where classified as *young*, while males after prime age were defined as *old*. A similar classification was made for the age the animals were removed from their natal group. Males removed from their natal group between the age of 3.5 and 6.5 years would have left their natal group at *normal* age [21,27,32]. Males that were removed from their natal group before the age of 3.5 were classified as *early*, while males removed after the age of 5.5 were classified as *late* migrants. For one male, information on the age he was removed from this natal group was lacking. This missing data point was filled with the average age (3.9 years) at which the males in our sample were removed from their natal group. Of the 26 introductions with *early* males, 22 were with males that were raised in peer-groups. These peer-reared males were generally introduced to a group during the transition period from the old single-mating system (i.e. individually housed females with infants being transferred to peer-groups after infancy) to the current naturalistic breeding groups at the BPRC between 1996 and 2001 [40], or concerned males from other research institutes with ‘standard’ breeding conditions (i.e., peer-groups formed following infancy) introduced into BPRC groups to increase genetic variability in the colony. Next, the number of matrilines, i.e. a group of animals that descended from the same female ancestor, was divided into two categories; groups with 3 or fewer matrilines are considered to have *few* matrilines (i.e. similar to the natural number in macaques [12,59]), while the remaining groups had *many* matrilines. Moreover, we indicated whether there was at least one natal male aged three or more present in the studied group. Similarly, we used the presence or absence of pregnant females in our analysis. The number of females in a group, the percentage of lactating females in the group, and male body weight were the only continuous variables in our models. However, the number of females was logarithmically transformed to fit the assumptions of the model. Generally, males were weighted when they moved to the enclosure adjacent to the females. If this was not the case, we used the last known body weight of the male before the introduction. On average, the males were weighed 96 days (range: 0–359) before their introduction started. An overview of all different predictor variables, their categories and definitions, and their descriptives can be found in Table 1.

**Table 1:** The predictor variables used in this study, including the different categories, their definition, and their descriptives (N, average and SD).

Variable	Categories	Definition	Sample size	Average (1 SD)
Timing	Early	Sept.–Dec.	N = 25	n.a.^1^
	Late	Jan.–Aug.	N = 39	n.a.^1^
Male age	Young	≤6 years	N = 22	5.8 (± 0.7)
	Prime	7–12 years	N = 28	9.5 (± 2.0)
	Old	≥13 years	N = 14	16.0 (± 2.7)
Body weight	Cont.^2^	Body weight in kg	N = 64	11.1 (± 2.0)
Experience	Yes	Was already breeding male	N = 27	n.a.^1^
	No	New breeding male	N = 37	n.a.^1^
Natal age	Early	<3.5 years	N = 26	1.6 (± 1.2)
	Normal	3.5–5.5 years	N = 24	4.8 (± 0.6)
	Late	>5.5 years	N = 14	6.3 (± 0.9)
Females	Cont.^2^	Log10(number of females)	N = 64	7.4 (± 2.9)
Matrilines	Few	≤3 matrilines	N = 41	2.2 (± 0.8)
	Many	≥4 matrilines	N = 23	4.3 (± 0.8)
Pregnant females	Yes	≥1 pregnant females	N = 19	2.1 (± 1.2)
	No	0 pregnant females	N = 45	0
Lactation	Cont.^2^	% females with infant <1 year	N = 64	30.5 (± 24.5)
Natal males	Yes	≥1 natal males aged 3 or more	N = 20	1.8 (± 1.0)
	No	0 natal males aged 3 or more	N = 44	0

^1^ Non-numerical variable

^2^ Continuous variable

All these variables were used to predict introduction success and whether a male obtained a long-term stable position in the group, called *long-term stability* in the remainder of this paper. An introduction was successful when the male remained in the group full-time for at least 4 weeks [18]. Whether the male obtained a long-term stable position was determined based on the removal of the male from the group. If the male was long-resident and was removed from the group for management reasons (e.g. preventing inbreeding with adult daughters), the introduction was long-term stable. When the male was removed from the group due to a behavioural problem (e.g. severe aggression or wounding by or to females) the group was not long-term stable. Two males were still in their new group at time of writing, and already spent 2.7 and 2.8 years in their groups. Up to now, they did not cause or experience any considerable behavioural problems and are not expected to do so in the future. Therefore, we treat the introductions of these males as long-term stable.

### Statistics

We used stepwise backwards logistic regression models to identify the effect of the above-named measures on introduction success and on long-term stability. Three separate models were run to test for the effect of introduction timing, male characteristics, and group characteristics on either introduction success or long-term stability. Introduction success and long-term stability were entered as the dependent variables, each in three models, while the predictor variables varied (Table 1). In the first model, the predictor variable was whether the introduction took place in the breeding season (i.e. timing). In the second model, male age, body weight, experience, and the age a male was removed from his natal group (i.e. male characteristics) were entered as predictor variables. The third model contained the number of matrilines, the number of females, the presence of pregnant females, the presence of natal males ages three or more, and the percentage of females with an infant (i.e. group characteristics) as predictor variables. When combining any of two categorical predictor variables included in the same model (e.g. determining how many groups with pregnant females also contained natal males aged three or more), all combinations of the variables occur at least once. One combination occurred once, two combinations occurred three times, while all other combinations occurred at least 5 times. However, there is one exception; there are no experienced young males in our sample. Therefore, we ran an additional model on introductions of prime and old males only.

Model selection was based on the Akaike information criterion (AICc). In each step, the variable that resulted in the lowest AICc after removal was removed from the model, until removing additional variables did not lower AICc further. The remaining model was considered the best predicting model. However, not every variable in the model may be of equal importance (i.e. contribute to the best fit). We considered a variable an important predictor of introduction success or long-term stability if delta AICc was close to or larger than 2 when removing the factor from the best predicting model. When delta AICc is much lower than 2, the factor is not considered an important predictor of introduction success or long-term stability. After selecting the best predicting models, we calculated Chi-square and McFadden’s pseudo R^2^ [60] to estimate the variance explained by the model. Finally, post-hoc testing was performed to identify the direction of the effect, or which categories within the predictor variables differed from each other. This was done by including the single predictors in the linear regression models.

Analysis were done using R studio version 1.1.4, with significance level set to p≤0.05. Collinearity within the models was checked; the variance inflation factor (VIF) did not exceed 2.95. Moreover, we tested the interdependence of the predictor variables within the same model (see *Results* section). Figures were created using the *Effects* package and visualize the outcome of the best predicting model.

## Results

### Interdependence of the predictor variables

The interdependence between predictor variables within the same model were tested. When related factors are included in the same best predicting model, we will provide additional analysis to identify which factor if the main predictor. Male age was related to male body weight (ANOVA, F(2,61) = 4.209, p = 0.019), male experience (Chi-square test: χ^2^ = 26.221, p<0.001) and the age at which a male was removed from his natal group (Chi-square test: χ^2^ = 12.716, p<0.013). In fact, young males were less heavy than prime and old males (Tukey HSD, p = 0.014), and were never experienced. Moreover, early removal from the natal group was less often present in young males (14%) than in prime (46%) and old (77%) males. There was no difference in body weight between the experienced and inexperienced males (t-test: t = 0.857, p = 0.395), or between males that were removed from their natal group at different ages (ANOVA: F(2,61) = 0.074, p = 0.929). Finally, male experience was unrelated to the male’s rearing condition (Chi-square test: χ^2^ = 4.472, p = 0.106). Thus, male age was related to all other factors in our model, while all other factors were unrelated. Therefore, we ran our analysis on male characteristics twice, once with the entire sample and once with the young males excluded.

Next, the interdependence between the predictor variables in the model concerning group characteristics was tested. The number of females in the group was not related to the presence of pregnant females (t-test: t = 0.401, p = 0.690), the number of infants in a group (Pearson correlation test: R = 0.022, p = 0.865), and the number of matrilines in the group (t-test: t = 1.730, p = 0.090). In contrast, groups containing natal males aged three or more also contained more females (t-test: t = 2.571, p = 0.013). The presence of natal males aged three or more was not related to the presence of pregnant females (Chi-square test: χ^2^ = 0, p = 1.000), the presence of infants in the group (t-test: t = 0.341, p = 0.735), or the number of matrilines in a group (Chi-square test: χ^2^ = 0, p = 1.000). Moreover, the presence of pregnant females was unrelated to the presence of infants (t-test: t = 0.621, p = 0.539), and the number of matrilines in the group (Chi-square test: χ^2^ = 0, p = 1.000). Finally, there neither was a relation between the number of matrilines and the presence of infants (t-test: t = 0.160, p = 0.874). Thus, the group characteristics are independent of each other, except for the presence of natal males aged three or more and the number of females in a group.

### Introduction success and long-term stability

A new male was successfully introduced to a group during 77% (N = 49) of the 64 introductions. 77% (N = 38) of the successfully introduced males did not cause considerable behavioural problems in the group in the years after and were eventually removed from the group for management reasons (i.e. were long-term stable). Altogether, 59% (N = 38) of all introduced males were able to obtain a long-term stable position in their group (Table 2).

**Table 2:** The number and percentage of successful and unsuccessful introductions, and the introductions in which the males obtained a long-term stable position in the group.

Success	Introduction success (N = 64)		Long-term stability–(success only) (N = 49)		Long-term stability (N = 64)	
Yes	49	77%	38	77%	38	59%
No	15	23%	11	23%	26	41%

### Introduction success

**Fig 1:**
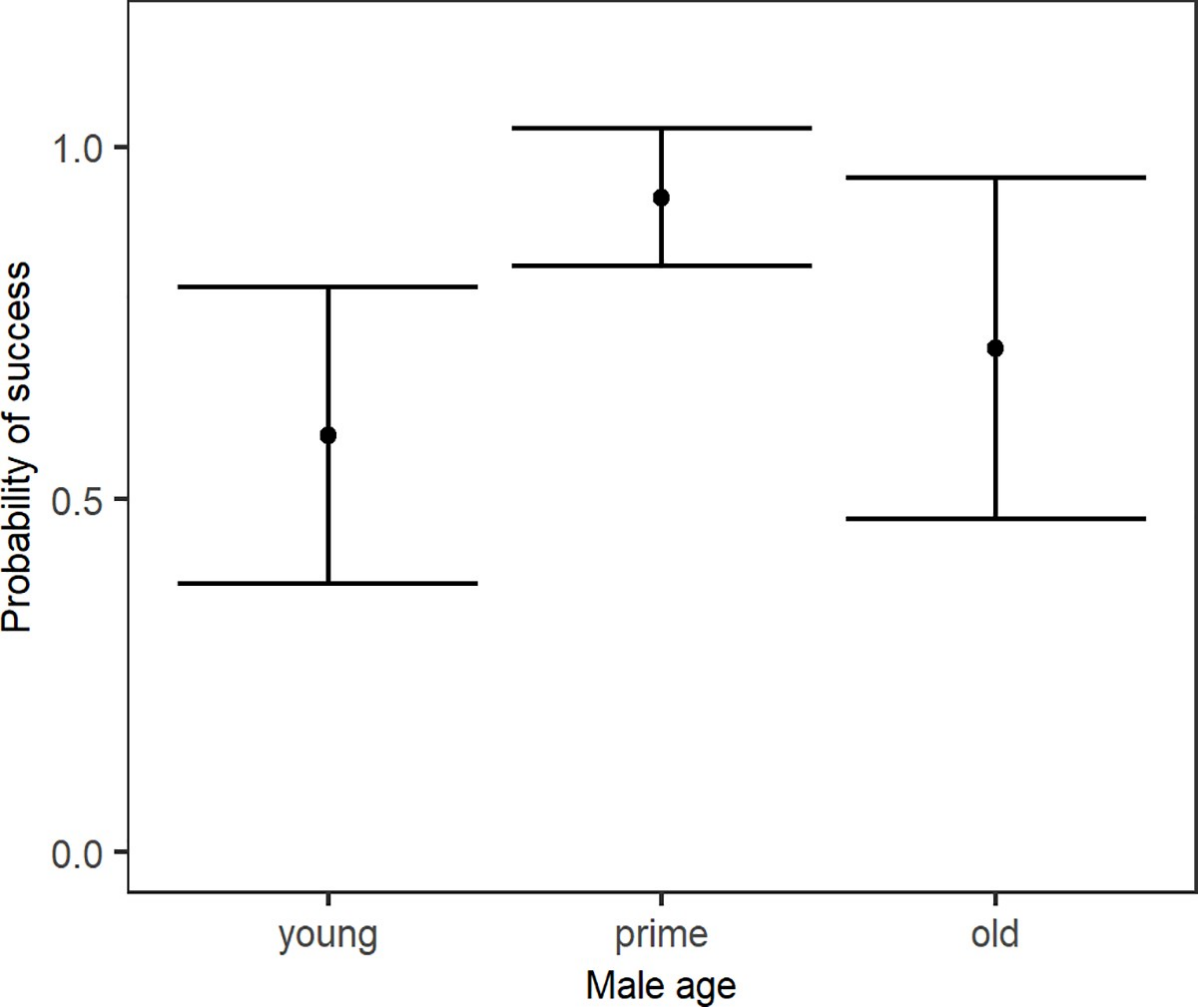
The effect of male age on introduction success (mean + 95% confidence interval).

**Fig 2:**
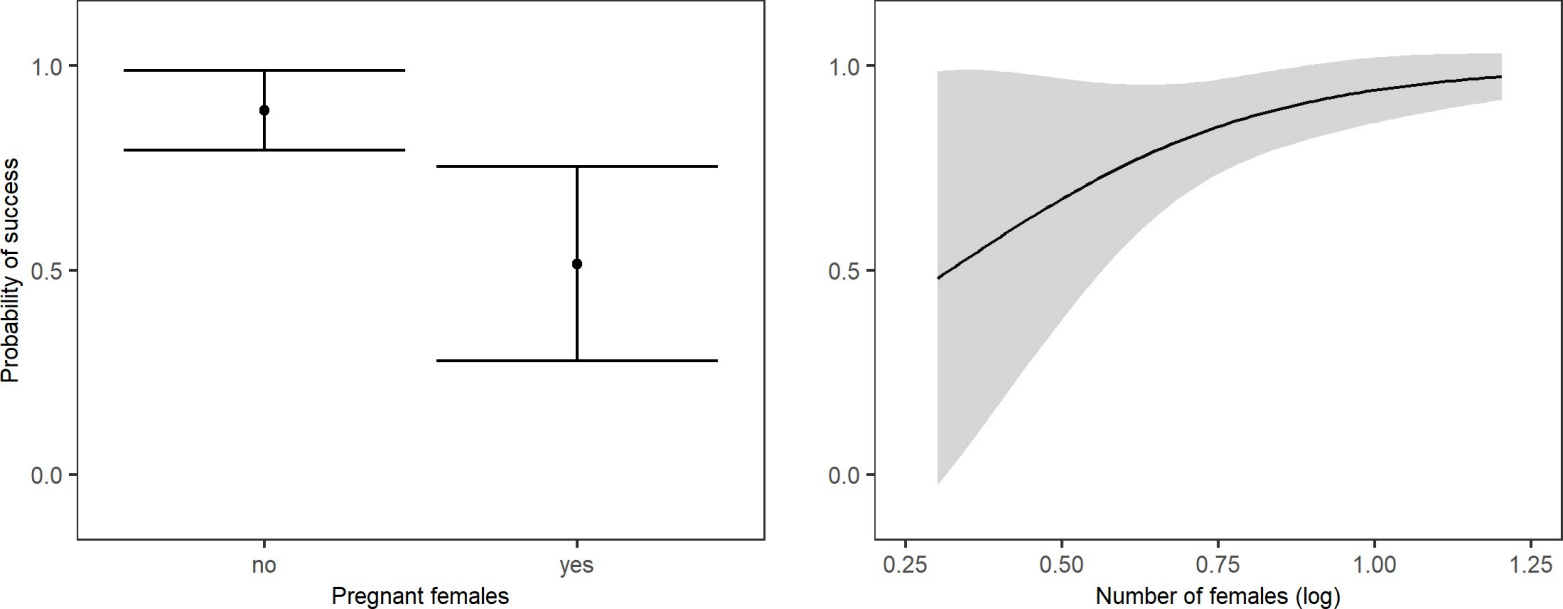
**The effect of the presence of pregnant females (left) and the number of females (right) in the group on introduction success (mean + 95% confidence interval)**.

We composed three separate models to test for the effect of seasonality, male characteristics and group characteristics on introduction success. First, introductions right before or early in the breeding season appeared to be more successful than introduction late in- or outside the breeding season (McFadden R^2^ = 0.046, χ^2^ = 3.206). Yet, delta AICc<2, indicating that the timing of an introduction is not a reliable predictor of success (Table A in S2 Appendix). Second, a model including the male characteristics age, experience and body weight predicted introduction success best (McFadden R^2^ = 0.190, χ^2^ = 13.226, Table B in S2 Appendix). Post-hoc analysis revealed that prime males were more successful than young males (McFadden R^2^ = 0.126, b = 2.197, z = 2.578), that heavier males had a higher chance of success (McFadden R^2^ = 0.081, b = 0.392, z = 2.157), and that more experienced males tended to be more successful (McFadden R^2^ = 0.061,b = 1.346, z = 1.906). However, there are no inexperienced young males in our dataset and young males are least heavy. Therefore, we ran the same analysis on a sample including prime and old aged males only. This results in a best predicting model with male age as the only predictor (McFadden R^2^ = 0.095, χ^2^ = 3.288, Table C in S2 Appendix). Additionally, when testing the effect of body weight on the success of young males only, the null model is the best predicting model (Table D in S2 Appendix). Taken together, this indicates that there is no effect of experience or body weight, and age is the most important male characteristic predicting introduction success. Indeed, delta AICc<1 when removing these factors from our model (Table B in S2 Appendix), implying no effect of experience and body weight on introduction success. We therefore consider the model with only age as a predictor the best predicting model, wherein young males are less successful than prime aged males (Fig 1). Third, in the model including group characteristics, the number of females in the group and the presence of pregnant females best predicted introduction success (McFadden R^2^ = 0.176, χ^2^ = 12.255, Table E in S2 Appendix). Post-hoc analysis revealed that introductions into groups with more females (McFadden R^2^ = 0.039, b = -4.060, z = -1.945) but without pregnant females (McFadden R^2^ = 1.116, b = 2.038, z = 2.921) were most successful (Fig 2). Taken together, introductions of males that had reached at least prime age, into groups with more females but without pregnant females were most successful.

### Long-term stability

**Fig 3:**
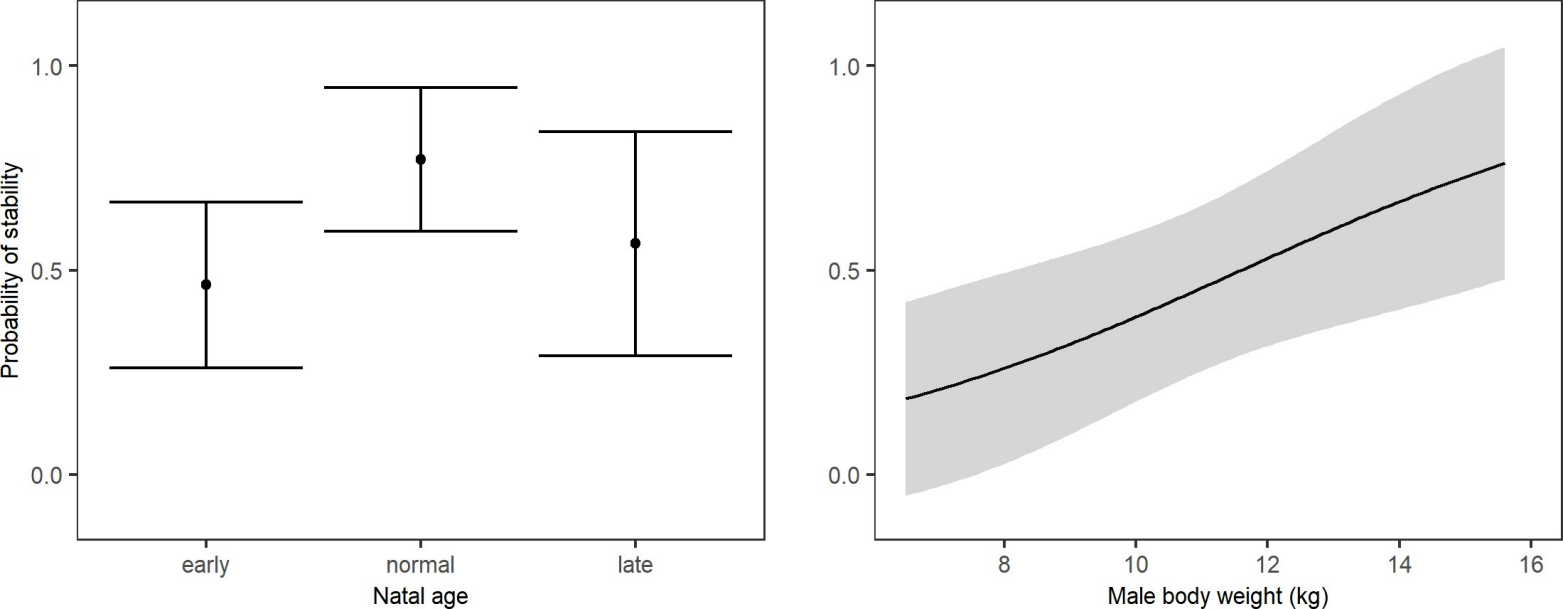
**The effect of the age a male was removed from his natal group (left) and male body weight (right) on long-term stability (mean + 95% confidence interval)**.

**Fig 4:**
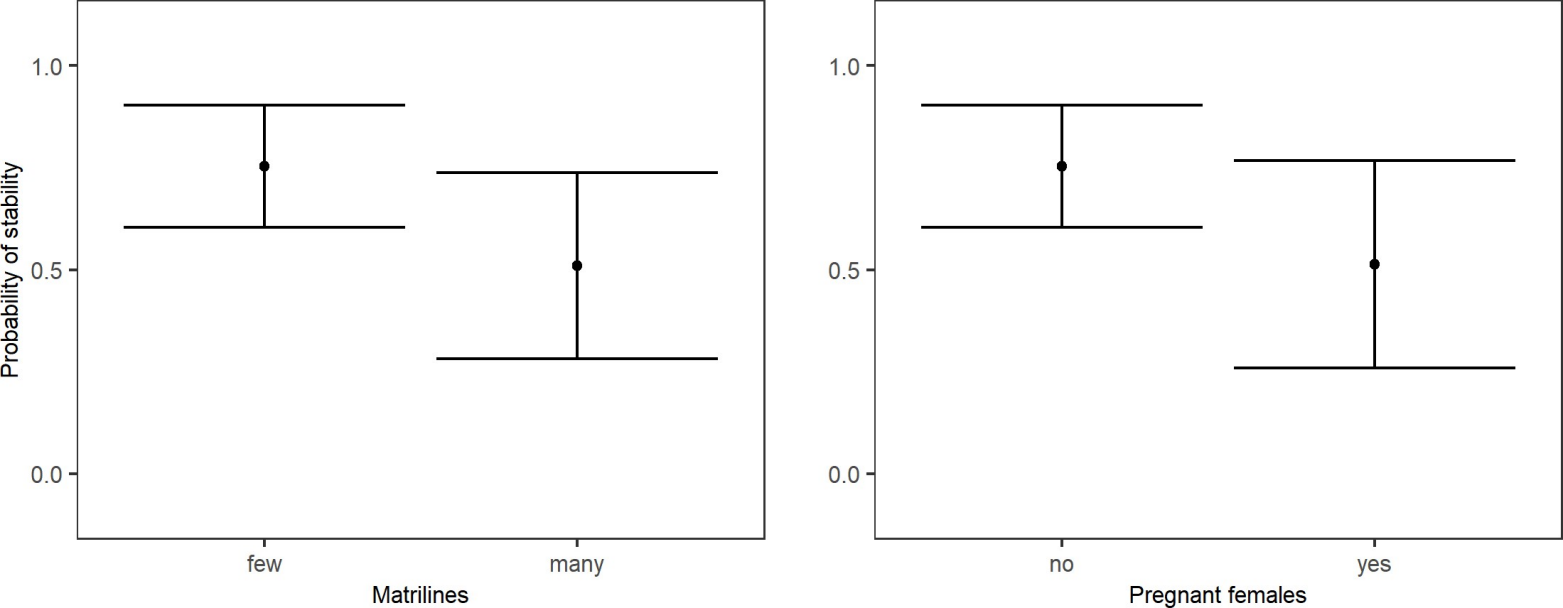
**The effect of the number of matrilines (left) and the present of pregnant females in the group (right) on long-term stability (mean + 95% confidence interval)**.

Three separate models were composed to test the effect of seasonality, male characteristics and group characteristics on long-term stability (including both successful and unsuccessful introductions). First, seasonality did not affect long-term stability (McFadden R^2^ = 0.015, χ^2^ = 1.282), as the null model was a better predictor of stability than the model including the timing of the introductions (Table F in S2 Appendix). Second, when focussing on male characteristics, long-term stability was best predicted by a model including male experience, the age a male was removed from his natal group, and body weight (McFadden R^2^ = 0.130, χ^2^ = 11.210, Table G in S2 Appendix). Post-hoc analysis revealed that introductions of males that were removed from their natal group at normal age were more often long-term stable than introductions with males that were removed from their natal group early (McFadden R^2^ = 0.051, b = -1.710, z = -2.407). Males that were removed late from their natal group did not differ from early removed males (b = -0.658, z = -0.895) or males removed at normal age (b = -1.052, z = -1.364) (Fig 3). The sample of males removed early from their natal group included peer-reared individuals, while the normal and late removed males concerned only group-reared individuals. To ensure the observed effect was not caused by peer-rearing only, we compared the long-term stability of peer-reared individuals with that of group-reared individuals. Yet, the null-model is a better predictor of long-term stability than the model including peer-rearing (Table H in S2 Appendix), implying that the observed effect of the age males were removed from their natal group is not caused by peer-rearing only. Introductions of heavier males were more long-term stable (McFadden R^2^ = 0.045, b = -0.288, z = -1.906) (Fig 3). Male experience was not an important factor in the model, as delta AICc<0.25 when removing male experience from the model (Table G in S2 Appendix). Finally, the model including group characteristics revealed that the number of matrilines and the presence of pregnant females in the group best predicted long-term stability (McFadden R^2^ = 0.083, χ^2^ = 7.177, Table I in S2 Appendix). Post-hoc analysis showed that introductions into groups with few matrilines (McFadden R^2^ = 0.043, b = 1.078, z = 1.937) and without pregnant females (McFadden R^2^ = 0.038, b = 1.065, z = 1.826) were more long-term stable (Fig 4). Delta AICc of the number of matrlines is close to 2, indicating that this factor is important for the model, while the contribution of pregnancy to the model is less clear as AICc≈1.4 for this factor (Table I in S2 Appendix). Taken together, introductions of heavy males that were not removed from their natal group at early age, into groups with few matrilines and without pregnant females were most likely to lead to long-term stability.

### Male experience

Male experience as a breeding male does not automatically certify future performance. Of the introductions of experienced males (N = 27) 89% were successful, while 67% was long-term stable. Moreover, males that were unsuccessful or unstable after their first introduction (N = 7) were always successfully introduced into another group (100%), and long-term stable after 71% of the introductions. Thus, a males’ performance during previous introductions cannot be directly translated to future introductions.

## Discussion

This study aimed to identify the factors affecting introduction success and long-term group stability of male rhesus macaque introductions. Introductions of males that reached at least prime age into groups with more females but without pregnant females were most successful. Long-term stability was highest after introductions of heavy males that were removed from their natal group when they were at least 3.5 years old, into groups with few matrilines and without pregnant females.

### Introduction success and long-term stability

Almost 77% of the studied introductions were successful, meaning that the male remained in the group full-time for at least four weeks. In the wild, males may fail to enter groups [13,17]; and unsuccessful immigration has even been reported in a wild group lacking adult males [61]. This shows that it is natural for males to fail in entering a group. However, it is not clear how high male immigration success in wild groups is. Male introduction success in captivity may vary from 0 to 100%, possibly depending on the timing of the introduction [20]. However, in this study [20] also the group composition differed between the successful and unsuccessful introductions: all introductions into female-only groups (N = 6) were successful and occurred during the breeding season; while all introductions into groups of females with dependent infants (N = 6) were unsuccessful and occurred outside the breeding season. In our study, group composition was more similar to that of the unsuccessful introductions, as the study groups consisted of adult females and their non-adult offspring. This indicates that 77% introduction success may be relatively high. Unfortunately, no other studies on male introduction success were found in literature. However, our view that 77% introduction success is relatively high is strengthened when comparing this to introduction success of pair-formation in macaques. Pair-formation only concerns two individuals and is therefore socially less complex that introductions in a group. Different studies reported pair-formation in macaques with success rates varying from 40 to 100%, with on average 85% success [3–8]. This implies that introducing a new male into an existing social group, while carefully managing the introduction, is almost equally risky as forming pairs. Although the current introduction success is reasonably good, understanding what contributes to introduction success may improve husbandry procedures even further.

About 77% of the males that were successfully introduced obtained a long-term stable social position in their new group. In unstable groups, the males were removed due to within group aggression (i.e. either male-female or female-male aggression). Social instability is associated with stress and injuries through increased aggression levels [22–25]. Moreover, removing the male from a group due to social instability, will lead to another risky introduction. Therefore, assessing social instability is crucial when aiming to improve animal welfare. In wild long-tailed macaques, up to 28% of the males may leave their group within several months after successful group entry [13,21]. These numbers are difficult to translate to our study as not all wild males obtained the alpha position in their new group, and male-male competition may have been at play. However, it illustrates that it is not unnatural for males to fail to settle in their new group after successful entry. To our knowledge, we are the first to report this process in captivity through focussing on long-term stability after male introductions. Similarly, one study focussing on pair-formation showed that 94% of initially successful pairs was compatible [4]. Altogether, this indicates that not all animals may be compatible, even after successful introductions, and that this phenomenon is natural. Therefore, groups should be closely monitored, even after the successful introduction of a male.

### Timing

Female receptiveness towards new males was expected to be higher right before and early in the breeding season; the time frame at which natural migration occurs [26–33]. However, the timing of the introductions did not affect introduction success or long-term stability. This contrasts with previous research on rhesus macaque introductions [20] and implies that female receptivity for males does not affect female hostility towards unfamiliar males. The main difference between this previous study and our study is the composition of the groups. The groups in our study were naturalistic, consisting of different matrilines, while the previously published study described male introductions into groups of unrelated females. Moreover, they did not focus on the male’s characteristics. Our results indicate that both male characteristics and the presence of matrilines are important for the outcome of introductions. Possibly, the new male’s characteristics and the group composition are most important in determining introduction success and long-term stability, and may thereby overrule the effect of timing.

### Male strength

Female resistance to new males may be less successful and cease sooner when stronger males are introduced. A male’s body weight can be used as a measure for strength, as high body mass corresponds to fighting ability in wild male crested macaques [10]. Heavier males in our study had higher long-term stability. This indicates that male strength plays a role during introductions. However, the relationship between male body weight and body condition in captivity is less straightforward than in the wild. Heavier males may be obese instead of stronger, as captive macaques may suffer from obesity [62]. Obese animals are less fit and may experience physical difficulties in conflicts with resident females. However, in our data set obesity is rare (BPRC data) and variability in body weight is likely more related to a male’s strength. Therefore, our results imply that stronger males are better able to obtain and maintain a position in a new social group, and only strong males should be selected for introductions.

In the wild, male strength may be greatest when males are at prime age. Indeed, younger males were less successful during introductions than prime aged males, while old males did not differ from young or prime aged males. This shows that males should not be introduced before they reached full adult strength. However, old males were as successful as prime aged males, while their body condition may be deteriorating. Possibly, captive primates are able to maintain their strength for longer than their wild counterparts due to food abundance in captivity. Therefore, older males may still be strong and successful during introductions. Altogether, our results indicate that heavy, prime aged and old males should be selected for introductions to increase the chances of introduction success and long-term stability.

### Male social history and experience

Males are expected to need social skills to obtain and maintain a position in a new group. Therefore, a male’s social history and experience may affect introduction success and long-term stability. First, the age at which a male was removed from his natal group affected long-term stability, but not introduction success. Males removed from their natal group before the age of 3.5 years were able to initially enter a group, but often failed to establish a stable social position on the long-term. Removing animals from their natal group too early often leads to inadequate social behaviour [38–40]. However, most studies reporting these effects compared peer-reared animals with animals growing up with their mother. Post-hoc analysis revealed that there was no difference in long-term stability between males that were peer-reared and male that grew up in naturalistic social groups. A statistical difference only emerged when taking the males removed from their natal group at early age together with the peer-reared males. Altogether, this indicates that it is important for males to be in their natal group at the onset of puberty. During this time, males may not only practice sexual behaviour, which drastically increases at puberty [63,64], but also learn social skills necessary to establish a stable social position in future groups. Therefore, males should stay in their natal group until they are at least 3.5 years old to fully develop their social behaviour. This corresponds to the age at which wild males naturally start migrating [21,27,32]. The current norm for weaning at research facilities is generally between 10–14 months [65]. This weaning age should be drastically increased to allow the animals to fully develop their social skills, thereby improving animal welfare and decreasing the risks of male introductions.

The presence of natal males aged three or more during introductions did not affect introduction success and long-term stability. The natal males were likely too small to affect male-male competition with the introduced adult males, which is a great cost wild males experience during migration [10,15,16]. Therefore, leaving males in their natal group until they are at least 3.5 years old will not affect future introductions into their natal group, while it improves the settlement of the males themselves in their future groups.

Second, a male’s experience as a breeding male did not affect introduction success and long-term stability. In the wild, males may change groups several times in their lives [21,41,42]. Males with experience in previous breeding groups could have gained social skills that enable them to better obtain a position in a new group. However, when selecting breeding males, their genetic representation in the breeding colony [66] should be taken into account. Experienced males already have offspring in the breeding colony, and may therefore be less preferred males for reintroduction. Therefore, inexperienced males are often preferred. Moreover, males that failed during their first introduction (i.e. unstable or unsuccessful) did not automatically fail during their second introduction. Thus, if males with valuable (i.e. underrepresented) genetics fail during their first introduction, introduction into another group can still be considered.

### Group composition

The composition of the group may affect a male’s ability to enter a group in the wild. First, introductions into groups with more females were more successful, while there was no effect of the number of females on long-term stability. This effect is surprising, as wild males do not select their new group based on the number of females [13,26,27,29,34,44]. Moreover, female coalitions against new males [17,18,43] can be larger in groups with more females. Therefore, introductions into larger groups were expected to be less successful. Possibly, since the number of matrilines is independent of the number of females in a groups, larger groups are more stable because they consist of larger matrilines. Female primates within a matriline form close social bonds [67,68]. These bonds may buffer stress during introductions [22], and increase stability within a group [48]. Increased stability is, in its turn, associated with lower aggression levels from females to males [25], which may increase introduction success. Second, introductions into groups with fewer than four matrilines resulted in more long-term stable groups, than introductions into groups with many matrilines. Wild macaque groups may consist of 1–3 matrilines [12,59], while groups with a large number of matrilines are unstable [11,46]. Males likely experience difficulties in obtaining a stable social position in a group when the relationships between resident individuals are already unstable. Overall, our results indicate that males should be introduced in groups with many females divided over few matrilines. To obtain such groups, females should remain in their natal group during their whole life to support the formation of large matrilines, similar to the wild.

### Female reproductive state

Female reproductive state was expected to affect a female’s attitude towards new males, and thereby affect introduction success and long-term stability. Pregnant and lactating females were expected to show increased resistance during male introductions since they are at risk of infanticide [49,50]. Even though infanticide risk is generally low in rhesus macaques, infanticide has been observed in this species [69]. Indeed, introductions into groups containing pregnant females were less successful, and less long-term stable. This indicates that infanticide risk can have long-lasting effects on female resistance to new males, which fits with the notion that males may commit infanticide until an infant no longer suppresses its mother’s fertility [69–72]. Therefore, also females with dependent infants at the start of the introduction may be at risk of infanticide when a new male enters a group [49,50]. However, the presence of infants did not affect introduction success or long-term stability. This contrasts with previous studies in species with high infanticide risk [50,52,53]. Possibly, part of the infants present during the introduction were at weaning age, and their mothers’ fertility was no longer inhibited by the nursing. Male infanticide is only beneficial if a female becomes fertile sooner when her infant is killed [73,74], as would be the case for pregnant females. Therefore, the infanticide risk is higher for pregnant females compared to lactating females. Overall, introductions into groups containing pregnant females should be avoided, while the presence of lactating females does not affect the outcome of introductions.

In summary, necessary male introductions in naturalistic captive groups are potentially risky, and sometimes unsuccessful. Implementing information on the male’s natural preference to enter specific groups and female resistance into captive introduction management can enhance introduction success and long-term stability. We show that carefully managed introductions are often successful and long-term stable. Introduction management can be further improved through only introducing males after they reached prime age. Moreover, introduced males should be heavy and should have remained in their natal group for at least 3.5 years. Groups should consist of few large matrilines, and preferably not contain pregnant females. Overall, the more closely the group and the introduction mimic natural migration patterns, the higher the chances of introduction success and long-term stability. This fits with the idea that animal welfare can be optimized when husbandry and group management remain close to the animal’s natural situation. Therefore, naturalistic group housing and dynamics are important to ensure long-term stable captive primate groups.

## Supporting information

S1 Appendix (XLSX)

S2 Appendix (PDF)
