# Supplementary material for: Factors determining male introduction success and long-term stability in captive rhesus macaques
Source: PLoS One. 2019 Jul 17;14(7):e0219972. doi: 10.1371/journal.pone.0219972 (PMC6636842; doi:10.1371/journal.pone.0219972)
Supplement: S2 Appendix — (PDF) [file pone.0219972.s002.pdf]

Appendix S2

Factors determining male introduction success and long-term stability in captive rhesus macaques

Astrid Rox, André H van Vliet, Elisabeth HM Sterck, Jan AM Langermans, Annet L Louwerse

Table A The delta AICc during stepwise backwards model selection of the logistic regression model on the effect of timing on introduction success.

**Step 1**	
Excluded	*Δ AICc*
-	0
Timing	1.206

Table B The delta AICc during stepwise backwards model selection of the logistic regression model on the effect of male characteristics on introduction success.

**Step 1**		**Step 2**	
Excluded	*Δ AICc*	*Excluded*	*Δ AICc*
Natal age	-1.754	-	0
-	0	Age	0.160
Experience	0.646	Experience	0.353
Age	0.626	Body weight	0.794
Body weight	1.556		

Table C The delta AICc during stepwise backwards model selection of the logistic regression model on the effect of male characteristics on introduction success, when only including introductions of prime and old males.

**Step 1**		**Step 2**		**Step 3**		**Step 4**	
Excluded	*Δ AICc*	*Excluded*	*Δ AICc*	*Excluded*	*Δ AICc*	*Excluded*	*Δ AICc*
Natal age	-3.575	Body weight	-0.338	Experience	-0.297	-	0
Body weight	-0.36	-	0	-	0	Age	1.289
-	0	Experience	0.365	Age	2.391		
Experience	0.396	Age	1.664				
Age	1.087						

Table D The delta AICc during stepwise backwards model selection of the logistic regression model on the effect of male characteristics on introduction success, when only including introductions of young males.

Step 1		Step 2	
Excluded	*Δ AICc*	*Excluded*	*Δ AICc*
Natal age	-1.692	Body weight	-0.819
-	0	-	0
Body weight	0.378		

Table E The delta AICc during stepwise backwards model selection of the logistic regression model on the effect of female characteristics on introduction success.

Step 1		Step 2		Step 3		Step 4	
Excluded	*Δ AICc*	*Excluded*	*Δ AICc*	*Excluded*	*Δ AICc*	*Excluded*	*Δ AICc*
Natal males	-1.532	Matrlines	-1.325	Lactation	-0.223	-	0
Matrilines	-1.515	Lactation	-0.125	-	0	Females	2.186
Lactation	-0.260	-	0	Females	2.390	Pregnancy	7.513
-	0	Females	2.952	Pregnancy	7.128		
Females	2.402	Pregnancy	7.127				
Pregnancy	7.241						

Table F The delta AICc during stepwise backwards model selection of the logistic regression model on the effect of timing on long-term stability.

Step 1	
Excluded	*Δ AICc*
Timing	-0.719
-	0

Table G The delta AICc during stepwise backwards model selection of the logistic regression model on the effect of male characteristics on long-term stability.

Step 1		Step 2	
Excluded	*Δ AICc*	*Excluded*	*Δ AICc*
Age	-3.281	-	0
-	0	Experience	0.427
Experience	0.139	Body weight	1.980
Body weight	1.298	Natal age	2.604
Natal age	1.571		

Table H The delta AICc during stepwise backwards model selection of the logistic regression model on the effect of peer-rearing on long-term stability.

**Step 1**	
Excluded	*Δ AICc*
Peer-rearing	-1.371
-	0

Table I The delta AICc during stepwise backwards model selection of the logistic regression model on the effect of female characteristics on long-term stability.

**Step 1**		**Step 2**		**Step 3**		**Step 4**	
Excluded	*Δ AICc*	*Excluded*	*Δ AICc*	*Excluded*	*Δ AICc*	*Excluded*	*Δ AICc*
Lactation	-1.945	Natal males	-1.395	Females	-0.944	-	0
Natal males	-1.368	Females	-0.608	-	0	Pregnancy	1.431
Females	-0.529	-	0	Pregnancy	1.658	Matrilines	1.868
-	0	Matrilines	1.685	Matrilines	2.618		
Pregnancy	1.605	Pregnancy	1.688				
Matrilines	1.696						
